# Rotatable high-resolution ARPES system for tunable linear-polarization geometry

**DOI:** 10.1107/S1600577517008037

**Published:** 2017-06-19

**Authors:** H. Iwasawa, K. Shimada, E. F. Schwier, M. Zheng, Y. Kojima, H. Hayashi, J. Jiang, M. Higashiguchi, Y. Aiura, H. Namatame, M. Taniguchi

**Affiliations:** a Hiroshima Synchrotron Radiation Center, Hiroshima University, Higashi-Hiroshima, Hiroshima 739-0046, Japan; b Graduate School of Science, Hiroshima University, Higashi-Hiroshima, Hiroshima 739-8526, Japan; c National Institute of Advanced Industrial Science and Technology, Tsukuba, Ibaraki 305-8568, Japan

**Keywords:** angle-resolved photoemission spectroscopy (ARPES), rotatable high-resolution ARPES system, linear polarization, condensed-matter physics

## Abstract

A rotatable high-resolution angle-resolved photoemission spectroscopy (ARPES) system has been developed to utilize tunable linear-polarization geometries on the linear undulator beamline (BL-1) at Hiroshima Synchrotron Radiation Center. By rotating the whole ARPES measurement system, the photoelectron detection plane can be continuously changed from parallel to normal against the electric field vector of linearly polarized undulator radiation. This polarization tunability enables us to identify the symmetry of the initial electronic states with respect to the mirror planes, and to selectively observe the electronic states based on the dipole selection rule in the photoemission process. Specifications of the rotatable high-resolution ARPES system are described, as well as its capabilities with some representative experimental results.

## Abstract 2

A rotatable high-resolution angle-resolved photoemission spectroscopy (ARPES) system that utilizes tunable linear-polarization geometries on the linear undulator beamline BL-1 at Hiroshima Synchrotron Radiation Center is described.

## 1. Introduction   

Macroscopic physical properties of solids are governed by microscopic dynamics of electrons. Angle-resolved photoemission spectroscopy (ARPES) is one of the powerful tools for directly studying the energy (ω) and momentum (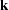) distribution of electrons, Fermi surfaces and energy gaps as a function of 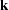 (Hüfner, 2003 ▸). Owing to significant improvements of instrumental energy and angular resolutions over the last two decades, one can now directly evaluate the electron self-energy, 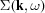 = 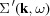 + 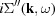, that includes all the information of many-body interactions. Here 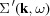 and 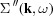 are real and imaginary parts of the self-energy, respectively. For instance, ‘kinks’ in the energy-band dispersion [peaks in 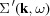] and ‘steps’ in the scattering rate [steps in 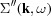] are the hallmarks of electrons coupled to a collective bosonic mode, and have been widely observed in various materials (Damascelli *et al.*, 2003 ▸; Hüfner, 2007 ▸).

In order to extract the self-energy, one needs to quantitatively analyze the line-shapes of energy distribution curves or momentum distribution curves. However, it is not always feasible to accurately resolve each of these spectral features in the line-shape analyses when several bands are crossing the Fermi level. To reduce the complexity, one can utilize the polarization property of incident photons to selectively observe the electronic states depending on their symmetry with respect to the mirror planes (Hüfner, 2007 ▸).

In this paper, we report our newly developed rotatable high-resolution ARPES system on the linear undulator beamline (BL-1) at the Hiroshima Synchrotron Radiation Center (HiSOR). This system allows us to continuously vary the polarization geometry from linear horizontal to linear vertical. The basic design and performance of the system and some representative polarization-dependent ARPES results are detailed. We demonstrate the significance of the linear polarization tunability for unravelling complex electronic states in solids.

## 2. Design and performance   

### 2.1. Dipole selection rule   

On the basis of the three-step model and the sudden approximation (Hüfner, 2003 ▸), the photoemission intensity 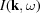 is given by
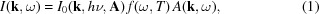
apart from the secondary electron backgrounds. Here, 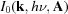 is proportional to the square of the dipole-transition matrix element 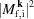, 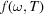 is the Fermi–Dirac (FD) function, 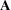 is the vector potential of the electromagnetic field (parallel to the electric field vector) and 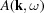 is the single-particle spectral function written as
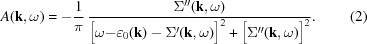
Based on Fermi’s golden rule, the dipole matrix element for transition from an initial state 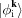 to a final state 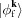 can be written as
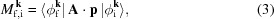
where 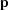 = 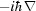 is a momentum operator. In order to fully utilize the selection rule, let the photoelectron detection plane lie in the mirror plane of the crystal (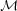), and let the vector potential 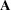 be in-plane or normal to 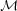, which are, respectively, referred to as the parallel 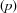 and senkrecht 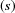 polarization geometries.

The non-vanishing photoemission intensity is only realised if the entire matrix element 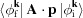 is a symmetric or even function with respect to the symmetry operation to 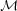. The final state 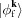 is set to be even with respect to 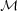 assuming that photoelectrons are free-electron-like and detected in 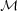 (Hermanson, 1977 ▸). Thus, the initial state 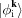 and the dipole operator 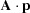 must have the same symmetry with respect to 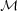 (Eberhardt & Himpsel, 1980 ▸). The non-relativistic dipole selection rule can be summarized as follows,
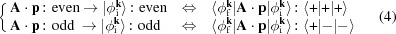
where 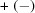 stands for the even (odd) function with respect to 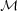. The symmetry of the dipole operator 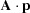 depends on the polarization direction of the vector potential 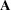: if 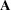 is parallel (perpendicular) to 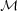, then 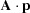 has an even (odd) symmetry. Therefore, the detectable initial state is limited to even (odd) symmetry with respect to 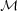 in the *p* (*s*) polarization geometry, indicating that one can selectively observe the even or odd initial electronic states by changing the polarization geometry. Hence, the dipole selection rule can be used to disentangle complex electronic states by reducing the number of detectable electronic bands according to their symmetry. In order to fully utilize the linear-polarization property, we have developed a high-resolution rotatable ARPES system as described below.

### 2.2. Rotatable ARPES system   

**Figure 1:**
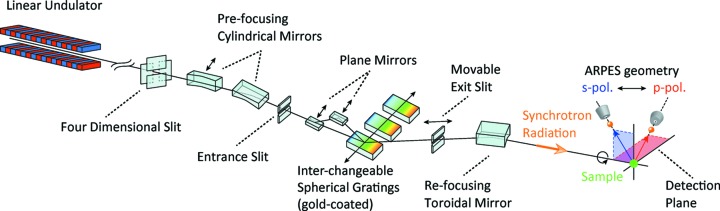
Schematic layout of the rotatable ARPES geometry and the linear undulator beamline BL-1 at HiSOR; the synchrotron radiation is linearly polarized in the horizontal plane. The detection plane of photoelectrons can be varied around the undulator light path by rotating the whole ARPES system, realising any *p*, *s* or arbitrary polarization geometry against the fixed horizontal polarization.

Fig. 1 ▸ schematically shows the layout of the linear undulator beamline (BL-1) of HiSOR (Shimada *et al.*, 2001 ▸), where we installed the new rotatable ARPES system. The linear undulator provides synchrotron radiation in the vacuum ultraviolet to the soft X-ray region (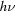 = 26–350 eV) with a photon flux of >1 × 10^11^ photons s^−1^, and the electric field vector lies in the horizontal plane. In order to change the polarization geometry against this fixed polarization direction, the photoelectron detection plane should be rotated around the undulator light path (Berrah *et al.*, 1999 ▸; Ali *et al.*, 2012 ▸).

**Figure 2:**
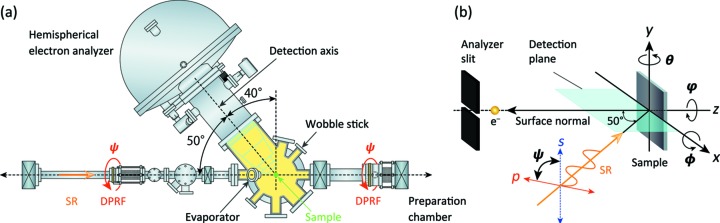
(*a*) Schematic top view of the rotatable ARPES system in the *p*-polarization geometry. The measurement chamber equipped with a hemispherical electron analyzer can be rotatable around the light path (0 ≤ Ψ ≤ 90°) by using two differentially pumped rotary feedthroughs (DPRFs). (*b*) Schematic view of the ARPES geometry, where the *p*-polarized light is incident on the photoelectron detection plane that is equivalent to the mirror plane of the sample. The polar, tilt and azimuthal angles of the sample are denoted as θ, ϕ and φ, respectively.

**Figure 3:**
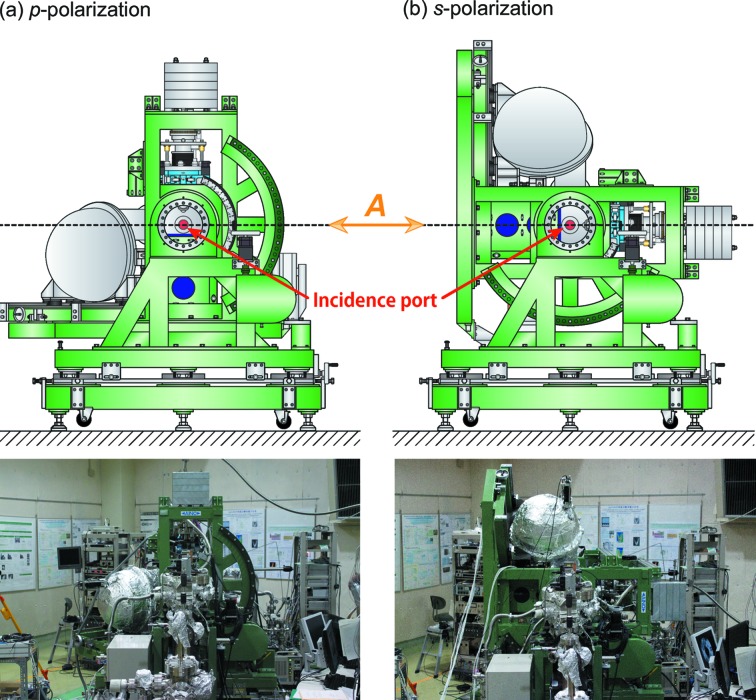
Schematic side view (upper) and photograph (lower) of the rotatable ARPES system for the (*a*) *p*- and (*b*) *s*-polarization geometry.

Fig. 2(*a*) ▸ shows a schematic top view of the rotatable ARPES system in the *p*-polarization geometry, where the entrance slit of the electron analyzer and the electric field vector of the incident linearly polarized light lie on the same plane. The sample surface normal is parallel to the electron analyzer’s lens axis in the *p*-polarization geometry [see Fig. 2(*b*) ▸], and the incidence angle of the undulator radiation is 50° relative to the lens axis. Rotation of the whole ARPES system is possible using two differentially pumped rotary feedthroughs (DPRFs) of ICF70 and ICF114 size, which were installed to sandwich the measurement chamber equipped with the hemispherical electron analyzer (VG-SCIENTA, R4000). The rotational angle (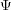) can be continuously changed by a computer-controlled stepping motor, allowing *p*, *s* or arbitrary linear polarization geometries from 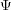 = 0° to 90°. Note that we can change the polarization geometry from *p* to *s* (or *vice versa*) within about 5 min, maintaining the ultrahigh vacuum (10^−9^ Pa range) in the measurement chamber. The 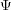 rotation can be regarded as the rotation of the electric field vector from *p* polarization (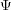 = 0°) to *s* polarization (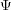 = 90°). The rotational mechanism can be more clearly seen in schematic side views and photographs of the rotatable ARPES system as shown in Fig. 3 ▸. In our system, the electron analyzer and other vacuum components were fixed to a specially developed rotatable frame (Aino Sangyo Co.), which is distinct from previous rotatable systems that adopted a pair of special DPRFs to sustain the weight of the electron energy analyzer (Berrah *et al.*, 1999 ▸; Ali *et al.*, 2012 ▸).

In Fig. 2(*b*) ▸, the sample orientation can be defined by the polar (θ), tilt (ϕ) and azimuthal (φ) rotations of the sample around the *y*, *x* and *z* axes, respectively. To manipulate these rotations *in situ*, we have developed two types of high-precision multi-axes manipulators with a liquid-helium-flow cryostat (R-Dec Co. Ltd, i-GONIO LT) (Aiura *et al.*, 2003 ▸). One type, a five-axis (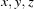 with θ and φ) manipulator, provides independent θ and φ rotations of the sample (0 ≤ θ ≤ 360° and −110° ≤ φ ≤ 110°), and can be cooled down to 6 K. The other type, a six-axis manipulator, provides an additional φ rotation (−20° ≤ ϕ ≤ 40°), which can cool the samples down to 15 K. Both manipulators were thus designed to provide full azimuth-maps (±90°) of the Fermi surfaces (FSs), which is indispensable for unambiguously defining the symmetry properties of the electronic states with respect to 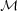.

All the motions/rotations of the samples as well as the chamber rotation (*x*, *y*, *z*, θ, ϕ, φ and 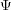) can be driven by computer-controlled stepping motors. For the remote control of all stepping motors and ARPES measurements, we have developed control software based on *LabVIEW* (National Instruments) using the functions named ‘SESWrapper’ provided by VG-Scienta. Accordingly, one can effectively and automatically acquire seamless ARPES data sets for the FS mapping, which allows a precise determination of sample orientations in a short time. Note that the software was developed to allow mappings with any axes combination as well as linear compensations of the sample positions (*x*, *y* and/or *z*) while angle-mapping (θ, ϕ, φ and/or 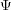) if needed.

**Figure 4:**
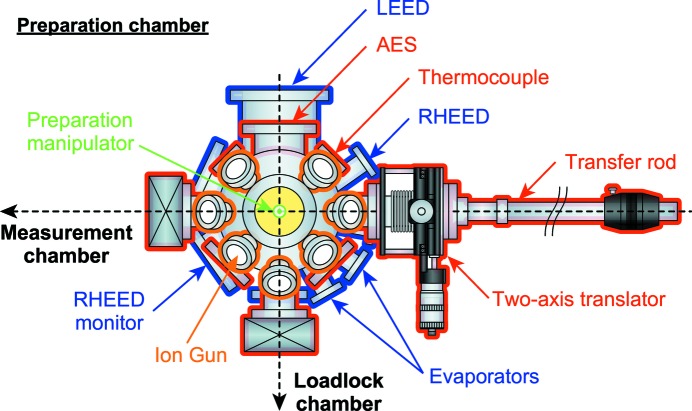
Schematic top view of the preparation chamber, where the height level is represented by, in order of descending height, orange, red and blue.

Fig. 4 ▸ illustrates a schematic top view of the preparation chamber, which is composed of three levels in height as indicated by colors: sample preparations can be performed at the lower two levels (red and blue), and sample transfer at the middle level (red). The chamber is equipped with a four-axis (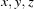 with θ) preparation manipulator with a liquid-nitrogen cryostat. We have developed a sample-holder-heating stage for direct current or electron-bombardment heating, and its temperature can be monitored by thermocouple or pyrometer. The sample position can be properly adjusted by the manipulator for ion sputtering, low-energy electron diffraction (LEED), Auger electron spectroscopy (AES), reflection high-energy diffraction (RHEED) and electron beam evaporators for the deposition of metals.

With the above-mentioned setup, a clean surface of single-crystalline metals or semiconductors can be obtained by ion sputtering and/or annealing at high temperatures up to ∼2500°C. Annealing in O_2_ as well as H adsorption by gas cracker are also possible. In addition, clean single-crystalline thin films can be fabricated by monitoring RHEED intensity oscillations to control their thickness. Note that one can obtain a clean surface of single- (poly) crystalline samples by cleaving (fracturing) using a wobble stick in the measurement chamber, and, further, can deposit metals (*e.g.* Li, Na, K, Cs, Au) on the sample surface from diagonally upward 45° by the evaporator, as shown in Fig. 2(*a*) ▸.

### 2.3. Energy resolution   

**Figure 5:**
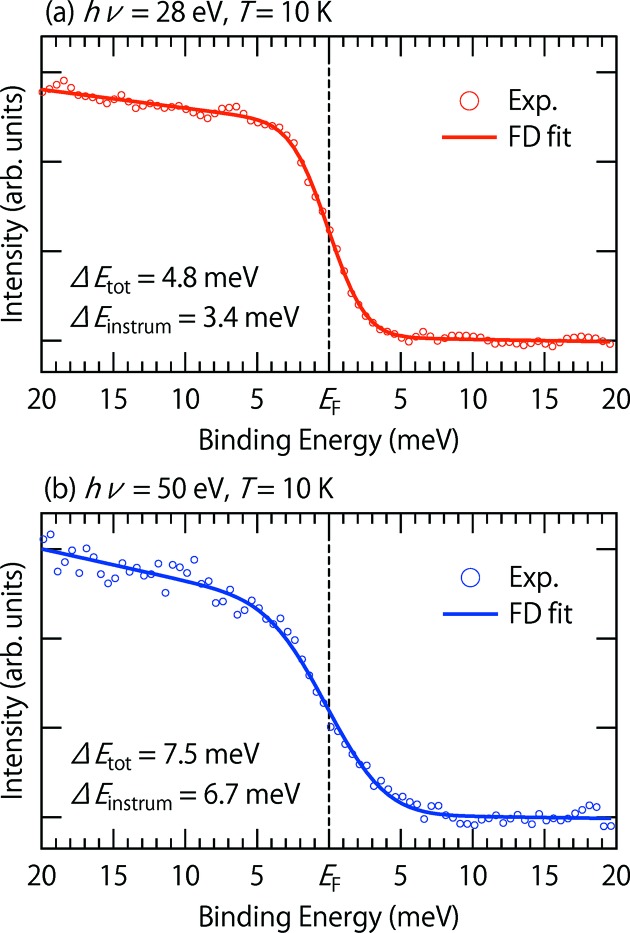
Angle-integrated photoemission spectra from evaporated polycrystalline gold taken at 10 K with a photon energy of (*a*) 28 eV and (*b*) 50 eV (open circles), where solid lines are obtained by curve fitting using the FD function convolved by a Gaussian.

Figs 5(*a*) and 5(*b*) ▸ show angle-integrated photoemission spectra from evaporated polycrystalline gold (open circles) taken at 10 K with 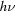 = 28 eV and 50 eV, respectively, with fits to the FD function (solid lines). The instrumental energy resolution 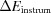, which is determined by the monochromator and electron-energy analyzer, and total energy resolution 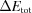 including the thermal broadening at 10 K, can be estimated as 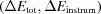 = (4.8 meV, 3.8 meV) for 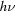 = 28 eV, and 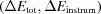 = (7.5 meV, 6.7 meV) for 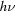 = 50 eV. Note that a practical working resolution would be optimized depending on the count rates taking into account the experimental purpose.

### 2.4. Polarization-dependent ARPES: Al(100)   

**Figure 6:**
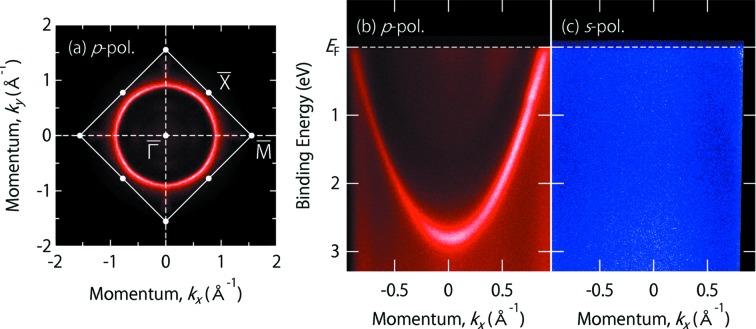
(*a*) Fermi surface of Al(100) taken with 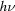 = 76 eV at 10 K in the *p*-polarization geometry, where white solid lines indicate the surface Brillouin zone of the face-centered-cubic (100) surface. (*b*) and (*c*) The ARPES intensity plot from Al(100) measured along the 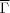
–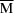 line with 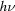 = 46 eV at 10 K in the *p*- and *s*-polarization geometry, respectively. The energy resolution was set at 25 meV for the Fermi surface mapping [panel (*a*)] and at 15 meV for high-resolution measurements [panels (*b*) and (*c*)]. After Jiang *et al.* (2011 ▸) for panels (*a*) and (*b*).

Aluminium is the textbook example of a trivalent nearly free-electron metal (Ashcroft & Mermin, 1976 ▸). Fig. 6(*a*) ▸ shows the FS mapping of Al(100) measured at 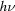 = 76 eV in the *p*-polarization geometry. One can clearly see a surface-derived circular FS centered at the 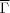 point of the surface Brillouin zone.

In Fig. 6(*b*) ▸, one can see a free-electron-like parabolic dispersion of the surface-derived state along the 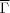
–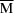 line using 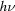 = 46 eV. By changing the polarization geometry, the parabolic dispersion completely disappears in the *s*-polarization geometry, as shown in Fig. 6(*c*) ▸. This is a natural consequence of the dipole selection rule, namely the observed surface state of Al(100) has an even symmetry under the reflection operation with respect to the (010) mirror plane. Note also that the complete suppression of the spectral intensity in the *s*-polarization geometry clearly indicates ∼100% linear polarization of the incident light.

### 2.5. Polarization-dependent ARPES: Sr_2_RuO_4_   

**Figure 7:**
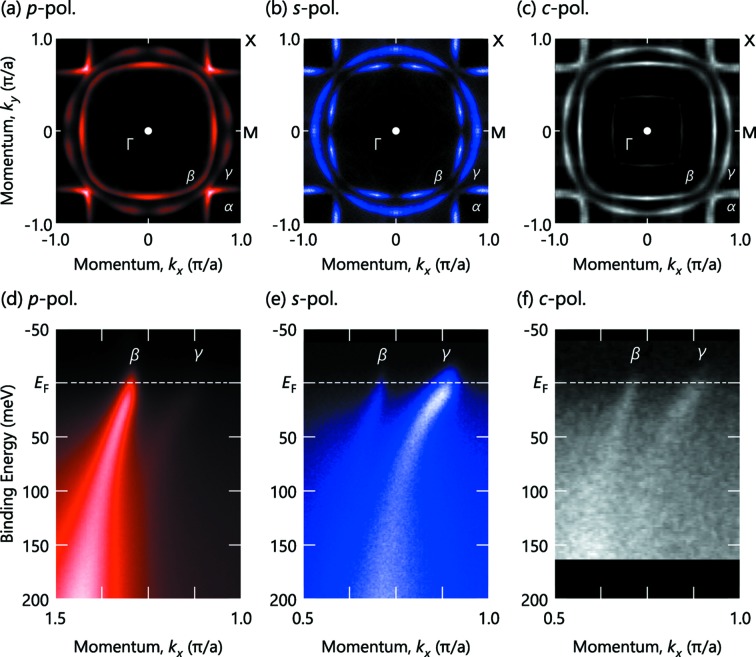
(*a*, *b*, *c*) Fermi surface of Sr_2_RuO_4_ taken at 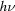 = 65 eV with *p*, *s*, *c* polarization, respectively. (*d*, *e*, *f*) The ARPES intensity plot from Sr_2_RuO_4_ measured along the 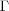
–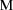 line with 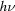 = 48 eV using the *p*, *s*, *c* polarization, respectively. The *p*-polarization data shown in panel (*d*) are taken at the second Brillouin zone because of the higher intensity of the β band than that in the first Brillouin zone, while the polarization dependence of the β and γ bands is essentially the same between them as seen in panel (*a*). Note that the energy resolution was set better than 30 meV and the measurement temperatures are 10 K for the *p*- and *s*-polarization data while the *c*-polarization data shown in panels (*c*) and (*f*) are recorded at 30 K and 20 K, respectively, at another undulator beamline (BL-28A, Photon Factory). After Iwasawa *et al.* (2010 ▸) for panels (*a*)–(*d*) and Iwasawa *et al.* (2005 ▸) for panels (*c*) and (*f*).

The single layer strontium ruthenium oxide (ruthenate), Sr_2_RuO_4_, is well known as a typical multiband superconductor (Mackenzie & Maeno, 2003 ▸), exhibiting three cylindrical FSs elongated along the *c*-axis. It cleaves well in the (001) plane where ARPES has revealed one hole-like FS (α) around the X point and two electron-like FSs (β and γ) around the Γ point (Damascelli *et al.*, 2000 ▸). Figs. 7(*a*)–7(*c*) ▸ show the FS maps measured at 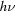 = 65 eV with *p*, *s* and circular (*c*) polarizations, respectively. The spectral intensities of the FS maps are significantly modulated for the *p* and *s* polarizations [Figs. 7(*a*) and 7(*b*) ▸], while not so dramatically changed for the *c* polarization [Fig. 7(*c*) ▸].

Along the 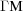 line, where a significant polarization-dependent intensity variation exists, we measured ARPES spectra at 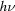 = 48 eV using the *p*, *s* and *c* polarizations [Figs. 7(*d*)–7(*f*) ▸]. With *c* polarization, one can observe both β (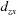) and γ (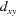) bands crossing the Fermi level, though they are only trackable down to a binding energy of 60 meV [Fig. 7(*f*) ▸]. The situation becomes completely different when measured in the *p*- and *s*-polarization geometries: the 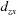 and 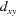 bands can be selectively observed in Figs. 7(*d*) and 7(*e*) ▸. This enables us to accurately determine the band dispersions over an extended energy region, and, furthermore, to make detailed self-energy analysis possible even in the complex electronic system (Iwasawa *et al.*, 2010 ▸, 2012 ▸; Hayashi *et al.*, 2013 ▸). The observed polarization dependence is consistent with the fact that the 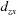 (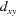) orbital is even (odd) symmetry under the reflection operation with respect to the (010) plane (Aiura *et al.*, 2010 ▸).

Meanwhile, one may notice that there exists weak but finite residual intensity of the 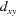 (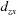) band even in *p* (*s*) polarization, which should vanish if the dipole selection rule strictly applies. Note that their intensities became almost the same at some excitation energies (Iwasawa *et al.*, 2010 ▸). Considering the very high linear polarization [∼100% based on the results from Al(100)] in both *p*- and *s*-polarization geometries in the present system as well as the precise sample alignment based on the FS maps, it is reasonable to assume that the deviation is derived from the mixture of the orbital characters due to the spin–orbit interaction (Iwasawa *et al.*, 2010 ▸; Aiura *et al.*, 2010 ▸). Having the high polarization as well as accurate sample alignment, the polarization-dependent ARPES can therefore provide an additional merit to prove the existence of orbital-hybridization by evaluating a deviation from the dipole selection rule.

## 3. Summary   

We have developed a rotatable ARPES system that enables us to continuously vary the angle of the electric field vector with respect to 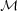, keeping the ultrahigh vacuum and low temperature. The advantages of linear polarization tunability were experimentally demonstrated in terms of the identification of the symmetry of the initial electronic states as well as the selective observation of the electronic states, which is indispensable for detailed study of the multiple band systems.
